# Rapid divergence of ecotypes of an invasive plant

**DOI:** 10.1093/aobpla/plu052

**Published:** 2014-09-01

**Authors:** Avik Ray, Rajasri Ray

**Affiliations:** 1 National Centre for Biological Sciences, Tata Institute of Fundamental Research, GKVK Campus, Bellary Road, Bangalore 560065, India; 2 Present address: Ashoka Trust for Research in Ecology and the Environment (ATREE), Royal Enclave, Sriramapura, Jakkur Post, Bangalore 560064, India, and Center for Interdisciplinary Studies (CIS), Basudha, Kolkata 700078, India; 3 Center for Ecological Sciences, Indian Institute of Science, Bangalore 560012, India

**Keywords:** Drift, ecotypes, gene flow, invasive plant, *Lantana camara*, niche, selection

## Abstract

Invasive species demonstrate rapid evolution within a very short period of time allowing one to understand the underlying mechanism(s). *Lantana camara*, a highly invasive plant of the tropics and subtropics, has expanded its range and successfully established itself almost throughout India. In order to uncover the processes governing the invasion dynamics, 218 individuals from various locations across India were characterized with six microsatellites. By integrating genetic data with niche modelling, we examined the effect of drift and environmental selection on genetic divergence. We found multiple genetic clusters that were non-randomly distributed across space. Spatial autocorrelation revealed a strong fine-scale structure, i.e. isolation by distance. In addition, we obtained evidence of inhibitory effects of selection on gene flow, i.e. isolation by environmental distance. Perhaps, local adaptation in response to selection is offsetting gene flow and causing the populations to diverge. Niche models suggested that temperature and precipitation play a major role in the observed spatial distribution of this plant. Based on a non-random distribution of clusters, unequal gene flow among them and different bioclimatic niche requirements, we concluded that the emergence of ecotypes represented by two genetic clusters is underway. They may be locally adapted to specific climatic conditions, and perhaps at the very early stages of ecological divergence.

## Abstract (precis)

Invasive species represent examples of rapid evolutionary change in a relatively short time period. *Lantana camara*, a well known invasive plant in the tropics and sub-tropics, is a suitable model system to study the mechanisms underlying its rapid spread and evolution. In order to understand the dynamics of *Lantana* invasion, we employed population genetics tools and found differential spread of two genetic varieties across the Indian landscape. Varieties also differ in terms of their climatic adaptation and gene flow, indicating possible local adaptation. Together, this may suggest that these varieties are divergent ecotypes at very early stages of differentiation.

## Introduction

Invasive species represent examples of rapid adaptive evolution in the contemporary time scale (Prentis *et al.* 2008). In a new habitat, selection can initiate diversification in a few generations, particularly when exposed to highly heterogeneous environmental regimes; drift can also accelerate quick changes in allele frequencies if the organism undergoes a bottleneck (Koskinen *et al.* 2002; Novak 2007). On the other hand, strong selective pressures could be alleviated by gene flow that keeps isolated populations connected by allowing movement of genes through outcrossing (Slatkin 1985; Garcia-Ramos and Kirkpatrick 1997; Hendry *et al.* 2001). One of the major limiting factors of gene flow among populations is geographic proximity, and that can be examined by testing isolation by distance patterns. However, earlier studies have demonstrated that, even in the face of gene flow, selection can be pervasively dominating to cause divergence (Kawecki and Ebert 2004). Divergent selection would act against migrant individuals so that only locally adapted ones would thrive and reproduce (Garrant *et al.* 2005; Postma and Van Noordwijk 2005).

In comparison, ‘isolation by environmental distance’ is a relatively recent approach used to explore the effects of environmental heterogeneity on genetic divergence. For instance, various bioclimatic (e.g. temperature, precipitation, etc.) and environmental variables (e.g. altitude, soil, etc.) tend to have a correlation with genetic divergence (Temunovic *et al.* 2012) that is suggestive of selection. However, in order to know a specie's diverse environmental requirements, one has to understand the complex association between species presence and environmental factors. Species distribution modelling or ecological niche modelling (ENM) explores this relationship through various algorithms and identifies suitable areas where potential populations could survive (Pearson 2010). While the application of niche modelling (species distribution modelling, SDM) to understand species ecology and distributions, and to design conservation planning is well established (Rodriguez *et al.* 2007; Urbina-cardona and Loyola 2008; Groot *et al.* 2009; Mateo *et al.* 2013), its implementation to uncover the role of physical and climatic factors in genetic differentiation has been realized very recently (Parisod and Christin 2008; Pease *et al.* 2009; Ortego *et al.* 2012; Temunovic *et al.* 2012). In order to understand invasion, niche modelling has been widely applied to delineate the future distribution potential (Zalba *et al.* 2000; Welk *et al.* 2002; Peterson 2003; Zhu *et al.* 2007; Petitpierre *et al.* 2012; Vaclavik and Meentemeyer 2012); however, studies integrating niche modelling with genetics to shed light on invasion dynamics are rare.

**Figure 1.**
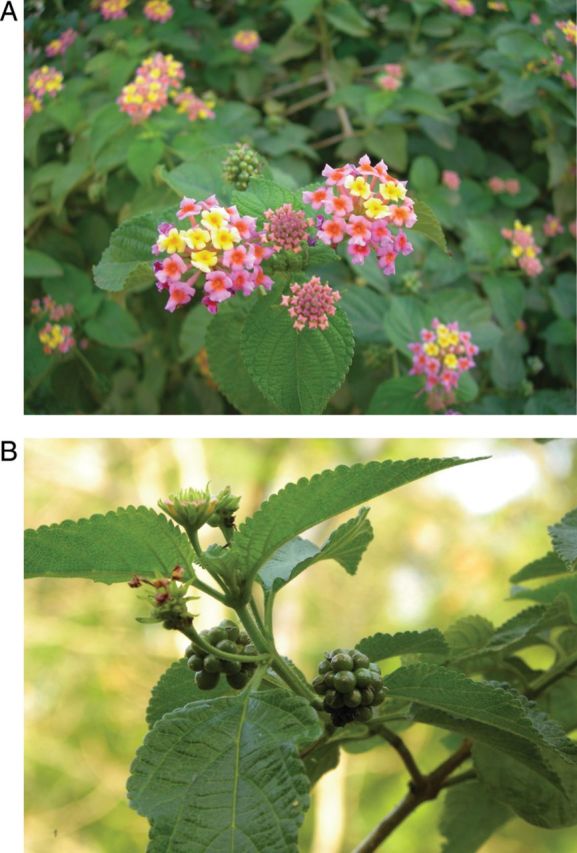
(A) Flowering and (B) fruiting twig of *Lantana camara*.

*Lantana camara* (henceforth *Lantana*) provides an interesting system which can be used to investigate such dynamics due to its recent history of invasion and establishment in India (Fig. 1A and B). A native of South and Central America, the plant was brought to India in the early 19th century as an ornamental plant. It was originally planted in the Indian Botanic Garden, Kolkata, and was subsequently introduced into northern (Dehradun) and southern India (Mysore) (Stirton 1978; Kohli *et al.* 2006). Eventually it escaped from gardens and gradually propagated and established itself across India. In the naturalized range, it often forms dense monospecific thickets or clumps (Swarbrick *et al.* 1998) and crowds out native species; perhaps thereby it invades or dominates an ecosystem (Duggin and Gentle 1998). The wider distribution of *Lantana* is attributed to its ecological tolerance and adaptability to various habitats (Broughton 2000; Day *et al.* 2003). In addition to that, its rapid spread over diverse biogeographic regions in a very short time span (∼200 years) has instigated several unanswered questions on its ecology, history, dynamics and their effects on the overall success of the species.

Previously, in order to understand invasion history, we have identified multiple introductions followed by gene flow and admixture (Ray and Quader 2013). In this paper, we aim to address the contribution of environmental selection and geographic distance to evolutionary divergence of the invasive plant *Lantana* in India. We have two broad objectives; first, unravelling the environmental requirements which effectively shape the spatial distribution of *Lantana* by niche modelling; and second, to determine whether or not environment can explain genetic divergence in concert with geography. In addition, we discuss the probable recent dynamics of *Lantana* and the long-term implications of these dynamics.

## Methods

### Sample collection, DNA extraction, microsatellite genotyping and summary statistics

**Figure 2.**
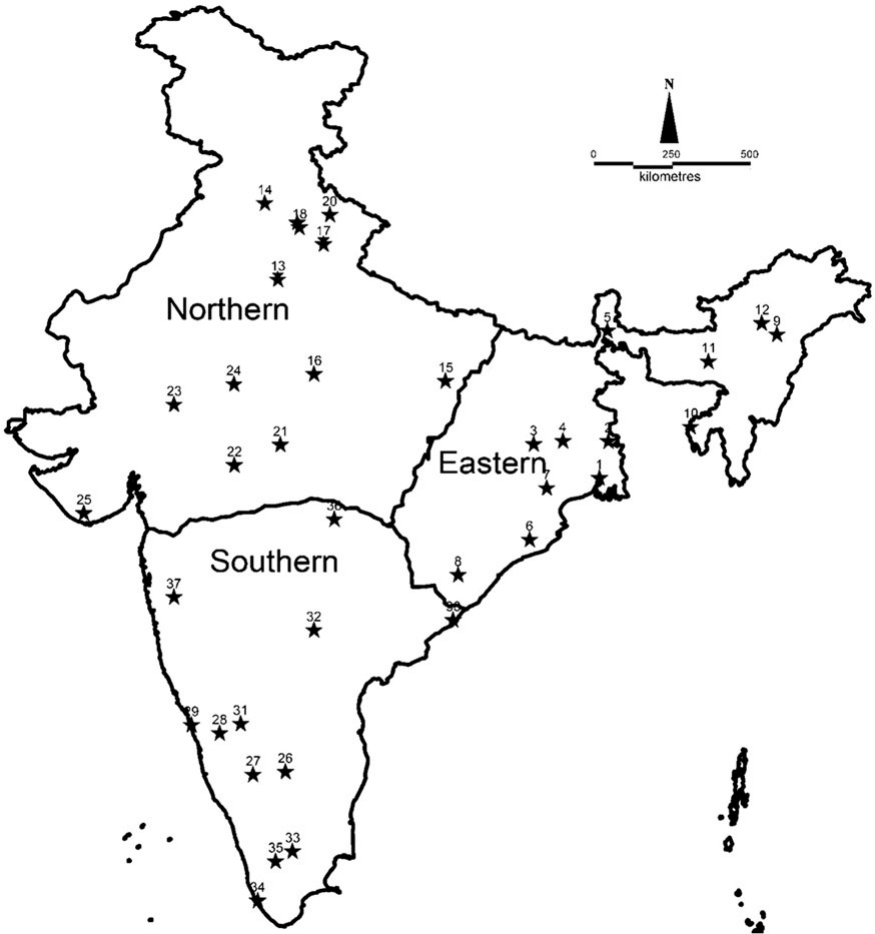
Geographic locations from which *Lantana camara* specimens were collected for this study. Populations (eastern, northern and southern) are demarcated by solid lines.

The literature mentions at least three independent introductions in Kolkata, Dehradun, and Mysore, in eastern, northern and southern India, respectively (Stirton 1978; Love *et al.* 2009). We collected 1–12 samples per site, for a total of 218 individuals in and around these primary introduction sites, as well as in other locations in those three general regions (Fig. 2; **see** **Supporting Information**). DNA extraction and microsatellite genotyping were carried out according to the method described by Ray *et al.* (2012).

In short, 218 individuals were genotyped with six microsatellites. The six microsatellite loci were highly polymorphic, with 9–19 alleles per locus yielding a total of 83 alleles **[see Supporting Information]**. The number of private alleles (i.e. those detected in only a single population) varied from 2 to 9 across loci, adding up to 27 private alleles in all. Pairwise *F*_ST_ values for microsatellites were very low (0.002–0.038) **[see Supporting Information]**. Standardized *F*_ST_ for microsatellites (scaled by maximum value, *F*_ST(max)_) showed moderate differentiation (0.056) (Ray and Quader 2013).

### Fine-scale spatial genetic structure

As our geographic population delimitation was somewhat arbitrary, we evaluated the fine-scale spatial genetic structure and the extent of gene flow by spatial autocorrelation analysis with GenAlEx ver. 6 (Peakall and Smouse 2006). Spatial autocorrelation does not require *a priori* population delimitation, but rather clusters individuals into different distance classes based on their geographic proximity. Analyses were based on pairwise genetic distance measures from the highly polymorphic microsatellite markers (Smouse and Peakall 1999). This method employs a multivariate approach to simultaneously assess the spatial signal generated by multiple loci. The autocorrelation coefficient (*r*) is a proper correlation coefficient, bounded by [−1, +1]. It provides a measure of the extent of genetic similarity between pairs of individuals whose geographic separation falls within the specified distance class. We performed the calculation several times with different bin widths for distance (e.g. 25, 50, 100, 200, 300, etc.), but we present data only for 100 since all bin widths demonstrated a similar pattern.

### Genetic clusters and their distribution

In the absence of strong population structure (*F*_ST_ = 0.056; Ray and Quader 2013), we sought to find out the number of divergent genetic clusters. We estimated the number of putative genetic clusters using a maximum likelihood-based method implemented in the program FLOCK (Duchesne and Turgeon 2009). This was done by repeatedly re-allocating similar multilocus genotypes to *K* populations (*K* is the number of potential genetic populations or genetic clusters) without using the geographical locations of individuals. Allocation decisions were based on the difference between the top two log-likelihoods (called log-likelihood difference or LLOD). A number of statistics detail the outcome of the ‘best’ run, i.e. the run with the highest global average LLOD score. Since each mean LLOD score is calculated with a high degree of precision, there is a high probability that the only two identical *K*-partitions will hit the same mean LLOD, and those runs are considered as ‘plateaus’ with length = *n*. Plateau length, *n* ≥ 6, is considered as good support for any partition. We ran the program five times for *K* = 2–20.

In order to understand the non-random distribution of genetic clusters, we developed a distribution map based on the spatial frequencies of the genetic clusters. In doing this, we first calculated the fraction of all individuals with ancestry (from LLOD values from FLOCK output) in each genetic cluster at each sampling site, and then we used that frequency for each cluster (clusters from FLOCK) to depict cluster distributions across India.

### Ecological niche modelling

Nineteen climatic variables with spatial resolutions of 1 km^2^ were downloaded from Worldclim dataset (www.worldclim.org) (Hijmans *et al.* 2005). To avoid redundancy and over-prediction, correlations among the 19 bioclimatic variables for 1200 random points from the study area were calculated to exclude the highly correlated ones (*r* > 0.7). In addition, five more variables (altitude, land cover, soil type, soil moisture, and solar radiation) were added to the modelling study based on the published literature (Taylor and Kumar 2012, 2013) (details of the data sources and correlation table are provided in **Supporting Information**). The layers, e.g. altitude, soil type, soil moisture, and solar radiation, were resized as per the study area and were resampled to 1 km^2^ to maintain uniformity among the raster layers.

Although *Lantana* is widely distributed throughout India, we tried to select fairly dispersed occurrence points to cover diverse ecosystems which are present in the country. A total of 123 occurrence points were finally selected based on field surveys and available literature. We applied the maximum entropy (Maxent ver. 3.3.3k) method for the modelling study due to its robustness and wider application for presence-only data (Phillips and Dudik 2008; Elith *et al.* 2011). Fifty per cent of the total data was used for model development and the remaining 50 % was used for testing the accuracy. The model was developed with default parameters set in Maxent. The model performance was evaluated by the area under the receiving operator characteristics curve (AUC) which ranges from 0.5 (random prediction) to 1 (perfect prediction). The logistic output had been selected for displaying the distribution map and the contribution of each variable was documented. Spatial autocorrelation of the model result was tested by Global Moran's I (Tiefelsdorf 2000). The final display was prepared based on the minimum training presence value as obtained from the model output. The same task was performed with genetic cluster-based occurrence points to develop two separate models for each cluster. To check the niche similarity between the clusters, a niche overlap test was conducted using ENM tools (Warren *et al.* 2010).

### Relationship between environmental and genetic distance

To account for variability in the response variable (genetic distance or divergence), we estimated the contribution from both geography (isolation by distance) and environment (isolation by environmental distance (ED) or adaptation) using partial Mantel tests and spatial (environmental) autocorrelation.

In order to conduct the partial Mantel tests, we grouped Cluster 1 (149 individuals) and Cluster 2 (53 individuals) into 32 and 19 populations, respectively (based on their spatial locations), and then calculated genetic, geographic and ED matrices likewise. Spatial autocorrelation is individual based, so genetic, spatial and environmental information were retrieved accordingly with 149 and 53 individuals for Clusters 1 and 2, respectively. In order to calculate ED, we used 10 selected bioclimatic and five environmental variables. Pairwise geographic and genetic distance matrices for Cluster 1 and 2 individuals were created using GenAlex (Peakall and Smouse 2006).

Partial Mantel tests determine the correlation between response and explanatory variables taking one at a time while controlling the other variable. The test was performed with the IBD web service using genetic and geographic distances with ED as the indicator matrix (Jensen *et al.* 2005). Significance was determined by 10 000 permutations.

Partial Mantel tests require prior delimitation of populations. However, the application of spatial autocorrelation to understand the influence of geography or environmental factors on genetic divergence allows no *a priori* assumption of population, but instead relies on individual-based information either spatial or genetic. The underlying rationale is to test whether or not gene flow is inhibited by ED, i.e. whether environmental selection is acting against migrant individuals and hence, only locally adapted pools of individuals are able to survive. We performed an autocorrelation analysis implemented in GENALEX ver. 6.2 (Peakall and Smouse 2006). The generated autocorrelation coefficient (*r*) provides a measure of genetic similarity (autocorrelation) between pairs of individuals whose ED falls within the specified distance class. We performed the calculation with Clusters 1 and 2, and repeated this several times with different bin widths for ED (at intervals of 0.1, 0.2, 0.3, 0.4, 0.5, etc.) to obtain consistent results.

### Comparison of gene flow scenarios between genetic clusters

Unequal gene flow between two genetic clusters may have a significant impact on the development of local adaptation. We compared different models of gene flow to investigate probable gene flow scenarios; the models vary in terms of the direction and magnitude of gene flow as follows: (i) Cluster 1↔Cluster 2 (bidirectional), (ii) Cluster 1→Cluster 2 (unidirectional), (iii) Cluster 1←Cluster 2 (unidirectional) and (iv) no gene flow. We used Migrate-n which applies the Bayesian inference to calculate probabilities of explicit population models by implementing coalescence theory (Beerli 2009). It calculates Bayes factor (BF) or log Bayes factor (LBF) values, which represent the ratio of the marginal likelihoods of two contending hypotheses or models. It also reports the effect of two different approximations of the log marginal likelihood on LBF and, therefore, the level of support for specific population models. We considered the Bezier approximation as this provides a better estimate of the marginal likelihood than the harmonic mean estimator (Beerli and Palczewski 2010). We used long chains = 1, long inc = 100, long sample = 10 000, burn-in = 10 000, replicates = YES: 5–50, random tree = YES, heating = YES with sampling at every 10th interval. We ran the program with 4, 9, 16 and 32 chains as in Beerli and Palczewski (2010), and noted the Bezier score and calculated LBF to choose the best model.

## Results

### Fine-scale spatial genetic structure

**Figure 3.**
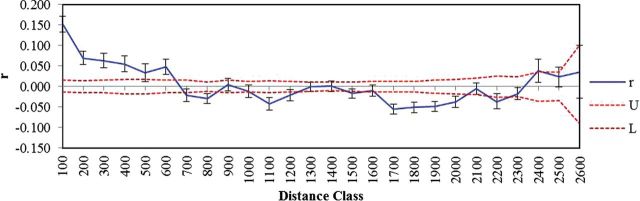
Correlogram showing the spatial correlation ‘*r*’ as a function of geographic distance class (100), 95 % CI about the null hypothesis of a random distribution and 95 % confidence error bars about ‘*r*’ as determined by bootstrapping. Upper (U) and lower (L) confidence limits of the 95 % CI about the null hypothesis of No spatial structure.

The autocorrelation coefficient (*r*) varied with increasing distance as well as among distance classes with different bin widths (e.g. 25–300). While comparing among distance classes with different bin widths, the smallest distance class size (50) yielded the highest autocorrelation coefficient (*r* = 0.196), and with increasing the bin widths (to 100, 200) the coefficient showed a gradual decline in magnitude (Fig. 3). In general, the coefficient (*r*) dropped steadily with increasing distance and beyond 500–600 the correlation coefficients were mostly non-significant, perhaps because of dearth of samples in those distance class groups. The moderate value of coefficients clearly indicates the presence of a local structure at fine spatial scales. Gradual decay of the correlation coefficient over increasing distance also ascertains isolation by distance patterns.

The spatial extent of the studied range is quite large, and choice of distance class may play a role in the estimation of structure (Peakall *et al.* 2003). Thus, despite taking one single distance class, we tested several distance classes of different bin widths (25–300, data only shown for 100) to understand the actual scale of action. We observed a clear trend of diminishing *r* values with increasing bin width. In general, we noted moderate spatial autocorrelation and isolation by distance up to a certain distance (500–600) after which it decays gradually.

### Genetic clusters

The most likely number of clusters was determined using maximum likelihood implemented in FLOCK. In FLOCK, after running five times, high values of plateau lengths (*n* ≥ 6) were obtained for *K* = 2, whereas very small plateau lengths (2 ≥ *n* ≤ 3) were obtained for *K* = 3, and no plateau was shown for *K* > 3. So, based on this support, i.e. high plateau length (i.e. *n* ≥ 6), we took *K* = 2 (hereafter Clusters 1 and 2) as the number of genetic clusters representing the variation. Many individuals were assigned entirely to a particular genetic cluster with high LLOD values [(±)9 to (±)19]. However, many had low-to-intermediate LLOD (−5 to +5) values which means that they lacked strong support for confident assignment into one group. So, it may further indicate shared ancestry of those individuals among multiple groups and they may be admixed as a result of outbreeding of multiple divergent source stocks.

Carefully looking at LLOD values in FLOCK runs, it appears that Cluster 1 is mostly compact, i.e. LLOD values do not vary much within that cluster. Contrary to that, the spread of LLOD values was (−5.74 to −19.02) greater in Cluster 2, i.e. there are two overlapping groups in Cluster 2, individuals with very high LLOD values (≤−12) and some with comparatively lower values (≥−12), which suggests that Cluster 2 is again sub-structured. Hence, we expected Cluster 2 to break into two groups when the number of clusters increased from *K* = 2 to *K* = 3. However, Cluster 2 almost remained intact while Cluster 1 divided into two, perhaps because the greater number of individuals in it provided high statistical support, or it may also be possible that the analysis is currently limited by the resolving power of the markers used.

**Figure 4.**
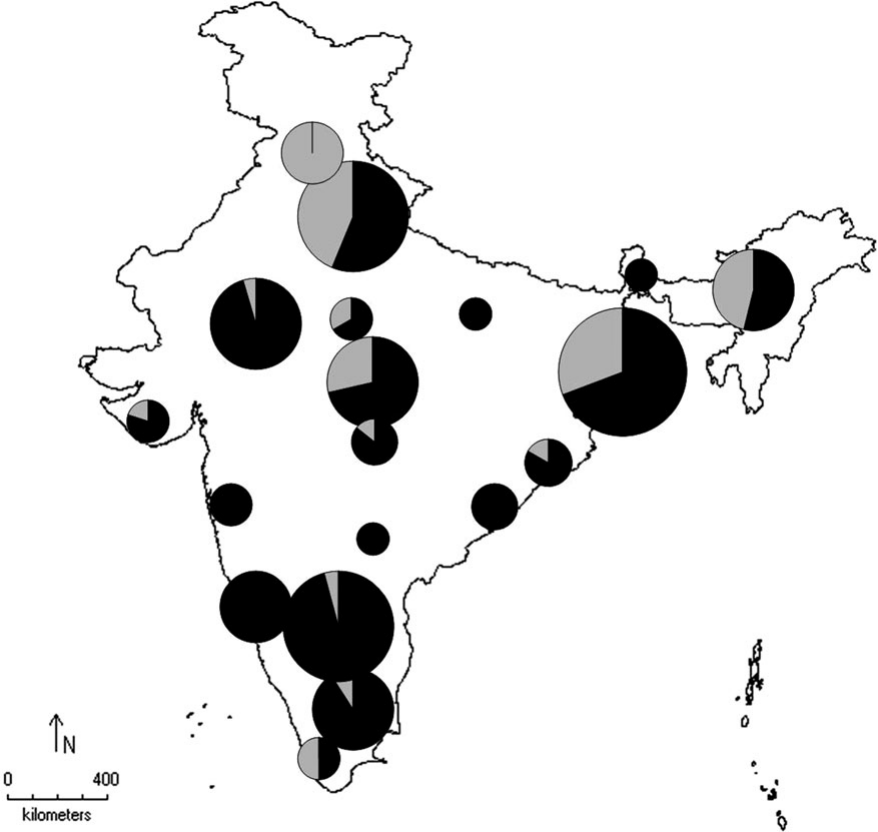
Distribution of clusters across India. Pie charts show percentage frequency of each cluster, and are proportional to the sample size at the corresponding sites. For better representation, some sites are combined with the nearest sites due to dearth of samples (less than five individuals). Clusters 1 and 2 are shown in black and grey colour, respectively.

Although Cluster 1 is almost relatively evenly distributed, it is present at higher densities towards the southern part of the range. The uneven distribution of Cluster 2 is very conspicuous; it is present in high frequencies in the northern region followed by the eastern, and is virtually absent from the southern area (Fig. 4).

### Niche modelling

**Figure 5.**
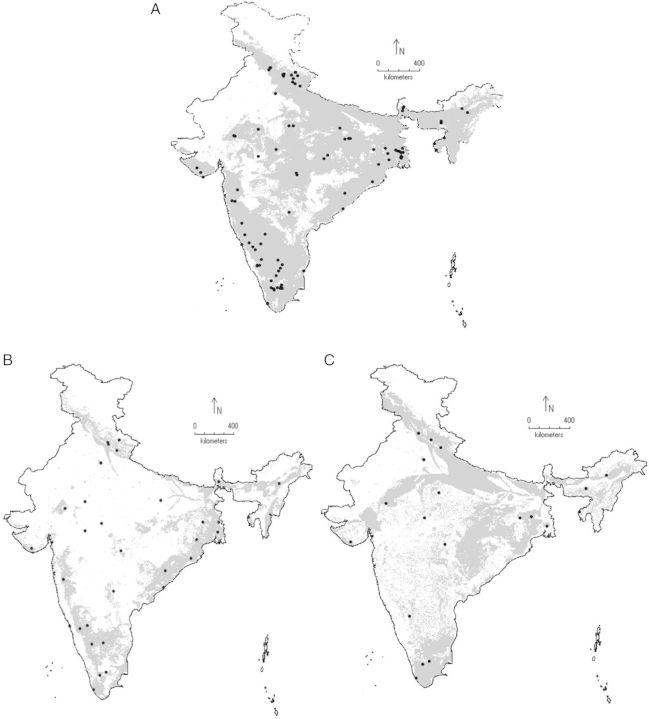
Maxent predictions of *Lantana* distributions for (A) the full dataset, (B) Cluster 1 and (C) Cluster 2. The binary distribution has been generated by using a minimum training presence value for the *Lantana* population (grey shaded areas are suitable for species growth). The niches were modelled using the 10 bioclimatic and five environmental variables.

The overall niche model has shown *Lantana*'s country-wide distribution, especially in the plain lands, i.e. the Gangetic plains, the foothills of Himalayas, the western and eastern coasts, southern and north-east India. Similarly, the binary distribution map based on minimum training presence values has shown the potential distribution area across the country except for the western desert and the high altitude zones in the Himalayas (Fig. 5A). The AUC of the test data was 0.869, indicating a good fitting of the modelled and current distribution records **[see Supporting Information]**, and model residuals showed weak spatial autocorrelation (Moran's *I* = 0.0752). The important variables were land cover, altitude and temperature seasonality (as per the jackknife plot for training gain, test gain and AUC). Models based on the genetic cluster data have shown slightly different distribution patterns. The Cluster 1 model (avg. train AUC = 0.965 ± 0.102, Moran's *I* = −0.1; **see Supporting Information**) shows high preference towards coastal areas, north-east India and the western Himalayan foothills (Fig. 5B). For the Cluster 2 model (avg. train AUC = 0.924 ± 0.131, Moran's *I* = −0.125; **see Supporting Information**), the central and eastern Indian plains were found to be moderate-to-highly suitable (Fig. 5C). However, distribution maps of genetic clusters show high niche similarity (*I* = 0.9701, *D* = 0.8108) indicating a near identical distribution pattern.

### Relation between the environment, geography and genetics

The partial Mantel test revealed a significant correlation between genetic and geographic distance in Cluster 2, whereas rest were all non-significant (Table 1).

**Table 1.** Correlations of genetic distance (Nei's *D*_S_) with geographic and environmental distance as measured with partial Mantel tests. Values in bold are significant (*Controlling for environmental distance. **Controlling for geographic distance).

Genetic group	Geographic distance*		Environmental distance**		Populations (sampling locations)
	*R*	*P*	*R*	*P*	
Cluster 1	0.0344	0.291	−0.0022	0.399	32
Cluster 2	**0.3047**	**0.008**	−0.0521	0.576	19

Spatial (environmental) autocorrelation clearly revealed fine-scale genetic structure. The autocorrelation coefficient (*r*) declined gradually with increasing distance from the origin as well as with increasing bin widths of different ED classes (e.g. 0.5–1.0) **[see Supporting Information]**. The highest magnitude of the coefficients at smallest distance class and then gradual decay over distance clearly indicates the presence of a fine structure and also ascertains isolation by ED pattern or local adaptation. When the magnitude of the structure was compared among various levels (i.e. whole dataset and cluster-wise), it revealed the highest coefficient in Cluster 2 (*r* = 0.345) at the first distance class while Cluster 1 yielded 0.243, perhaps indicating differences in the strength of selection.

### Gene flow scenarios

The various runs (differing chain lengths in heating and number of replicates) were consistent in terms of model comparison; however, the absolute values of LBF differed among runs. We obtained strong proof of unequal gene flow between clusters. Among the different models used to identify most probable scenario, unidirectional gene flow from Cluster 1 to Cluster 2 (Model 2) scored the highest followed by Model 1 (bidirectional), Model 3 (Cluster 2 to Cluster 1) and Model 4 (no gene flow) (Table 2). The high LBF value for Model 2 indicated very strong support for it and validated its acceptance (Beerli and Palczewski 2010).

**Table 2.** Comparison of various gene flow models (i) Cluster 1↔Cluster 2 (bidirectional), (ii) Cluster 1→Cluster 2 (unidirectional), (iii) Cluster 1←Cluster 2 (unidirectional) and (iv) no gene flow with respective Bezier lml and LBF scores showing the most acceptable model (in bold).

Model	Gene flow scenarios	Number of chains	Bezier lml	LBF	Model ranking
1	Cluster 1↔Cluster 2 (bidirectional)	4	−320 986.24	0	2
		9	−14 425.77	0	
		16	−65 440.76	0	
		32	−39 961.45	0	
2	**Cluster 1→Cluster 2 (unidirectional)**	4	−257 897.6	63 088	1
		9	−13 899.21	526	
		16	−58 376.55	7064.21	
		32	−32 404.83	7556	
3	Cluster 1←Cluster 2 (unidirectional)	4	−375 216.92	−54 230	3
		9	−16 762.03	−31 187	
		16	−84 643.62	−19 202	
		32	−45 745.13	−5783	
4	No gene flow	4	−543 022.54	−222 036	4
		9	−35 610.26	−21 184	
		16	−132 631.46	−67 190	
		32	−76 136.09	−36 174	

## Discussion

In recent years, the application of a multitude of algorithms to model ecological niche of a species or easy retrieval of climatic data has facilitated attempts to append environmental with genetic data, to determine inter-relationship in various statistical frameworks and to make inferences about dependence or its lack thereof (Knowles *et al.* 2007; Manel *et al.* 2010; Sork *et al.* 2010). As a result, highly polymorphic markers (e.g. microsatellites, AFLP) coupled with niche modelling have been employed to trace adaptive divergence (Knowles *et al.* 2007; Pease *et al.* 2009; Ortego *et al.* 2012). In some instances, tight correlations between phenotypes and genetic distance have also been inferred and attributed to environment-driven selection and incipient speciation (Funk *et al.* 2009; Ramírez-Valiente *et al.* 2010; Wang and Summers 2010).

Despite being effective model systems to examine adaptive evolution, invasive species are quite poorly studied, at least in terms of understanding the potential role of selection in their rapid spread. The patchy geographic distribution of genetic clusters of *Lantana* left much space to ask further questions. For instance, what are the reasons behind this distribution? In addition to drift, is selection driving the divergence and causing the patchy distribution? Is there any local adaptation? We addressed some of these questions by analysing molecular marker data along with niche modelling tools, and investigated the contribution of environmental selection and genetic drift to *L. camara* invasion in India.

### Effect of drift on gene flow

Spatial autocorrelation often reveals subtle spatial structuring at a local scale which cannot be identified by summary statistics like *F*_ST_ and its analogues (Peakall *et al.* 2003). The scale of gene flow uncovered in this way is related to the underlying scale of pollination and dispersal (Manel *et al.* 2003; Holderegger *et al.* 2010) and is shaped by the plant's mating system, seedling establishment and mortality, barriers to gene flow, and so on (Gonzales *et al.* 2010). Fine-scale structure is also influenced by population history, drift and selection (Heywood 1991). Tropical tree species mostly have clumped distribution, partly due to limited seed dispersal ability (Jones *et al.* 2005) and demonstrate high levels of spatial structure (Gonzales *et al.* 2010). At the other end of the size scale, herbs may also show high levels of structure at a fine scale, again due to restricted propagule movements (Torres *et al.* 2003). Invasive species, on the other hand, are expected to exhibit low levels of spatial structure because of their rapid pace of recent spatial expansion. The magnitude of local structure is likely to depend on the phase of their range expansion, the geographic scale of measurement, the proximity to neighbours, and their dispersal ability.

The presence of fine-scale genetic structure implies that the gene flow is not random and is constrained by distance. The prevalence of isolation by distance inferred by spatial autocorrelation is also in agreement with earlier conclusions (Ray and Quader 2013) and is robust since there is no *a priori* population delimitation. This suggests that populations are experiencing moderate gene flow over relatively short distances (i.e. <500–600), and low gene flow across longer distances. So, the current pattern of distance-limited gene flow may be implicated in the development of some local adaptation and can further stimulate adaptive divergence of geographically distant populations. Rapid short-distance pollination and dispersal can be attributed to the mobility of the birds and butterflies that pollinate *Lantana*, and the small-to-large mammals and birds that disperse the seeds (Johnson 2007). On the other hand, spread over a large spatial scale may require the formation of small satellite populations that serve as a source for secondary or tertiary spread. However, questions on long-distance gene flow of *Lantana*, its mode and extent, still remain.

### Environmental niche requirements

Global-scale modelling studies on *Lantana* have predicted its widespread distribution in India, perhaps due to broad geographic scale and lack of sufficient occurrence points in India (Bhagwat *et al.* 2012; Taylor and Kumar 2012). *Lantana* distribution models for Australia found that temperature (i.e. limiting low temperature, limiting high temperature) and soil moisture (i.e. soil drainage type) play key roles in its current and potential distribution pattern. Similarly in China, models were built using temperature, precipitation and elevation variables due to their importance in eco-physiological activities (Lüi 2011). In contrast, our model was developed based on the widely distributed occurrence points in India, which are expected to encompass heterogeneous environmental regimes, thus promising better predictive power than the previous models. The potential distribution map reflected its moderate-to-high country-wide distribution potential, except in the western desert and the northern Himalayan regions, and this is well supported by available literature as well as field observations (Thakur *et al.* 1992; Kannan *et al.* 2012). The pattern becomes clearer with a binary distribution map based on minimum presence points. The model revealed preference towards variables like temperature seasonality (Bio4), altitude and land-cover patterns which share a similarity with findings from elsewhere (Lüi 2011; Taylor and Kumar 2012, 2013). Grossly, the favourable areas have higher seasonality values in comparison with the non-favourable ones, indicating species tolerance to harsh climatic conditions, except in the western desert region as revealed in other studies (Broughton 2000; Day *et al.* 2003). Similarly, altitude plays an important role as the distribution almost discontinues near the Himalayan foothill area and different parts of central Indian highlands. However, the Western Ghats, one of the major biodiversity hotspots, is found to be moderately favourable for its spread as reported previously (Kannan *et al.* 2012).

Although the genetic clusters are moderately restricted in their spatial distributions (confinement is very distinct in Cluster 2) we did not observe any major differences in their potential distributions except in the Deccan plateau, and the central and eastern Indian highlands. These have encompassed slightly different areas with moderate niche similarity. The model based on Cluster 1 data has shown a greater contribution from land cover, soil type, temperature seasonality (Bio4) and altitude variables. In Cluster 2, precipitation (Bio16) has a major share along with Cluster 1 variables. The rapid spread of the species (both the genotypes) could be attributed to major land-use changes which have taken place in the country. It may be because of the degradation, fragmentation, and conversion of the forest lands creating favourable habitats in terms of higher light availability, moderate soil moisture, and other microenvironment parameters. Overlapping areas in the predicted niche space of the clusters are in stark contrast to our expectation. In a few other organisms, genetic clusters have demonstrated the ability to occupy unique and non-overlapping predicted niche areas (Pease *et al.* 2009; Lee and Mitchell-Olds 2011). Being native, these organisms have perhaps become locally adapted over much longer periods of time, so niche separation of the ecotypes/clusters appeared very distinct. In contrast, for an invasive species with only 200 years of demographic history in the new range, the climatic niche may be at a very early stage of differentiation.

### Isolation by ED and local adaptation

Phenotypic or genotypic correlations with environmental factors have long been regarded as signatures of selection (Endler 1986). However, the partial Mantel test was unable to track the effects of both environment and geography except in Cluster 1. In contrast, our previous results found isolation by distance patterns using Mantel test (Ray and Quader 2013). Distance-based methods are sometimes constrained by lack of statistical power and are not aptly able to explain the variance in response variables (Legendre and Fortin 2010). Likewise, a few studies have partially failed to find significant correlation with partial Mantel tests (Pease *et al.* 2009). However, spatial (environmental) autocorrelation analysis implies the presence of fine-scale genetic structure and non-random gene flow. Gene flow appears to be inhibited by ED, i.e. due to the presence of locally adapted genotypes or individuals. Selection is perhaps inhibiting migration by acting against non-locally adapted genotypes and ultimately favouring traits that would confer advantages under local environmental conditions (Garrant *et al.* 2005; Postma and Van Noordwijk 2005). The prevalence of non-random gene flow is also evident from a comparison of different gene flow models, i.e. one-way gene flow from Cluster 1 to Cluster 2 was the most supported model with the highest BF value. Local adaptation of Cluster 2 genotypes possibly has been restricting their migration away from regions with favourable environmental conditions. This scenario is supported by the higher autocorrelation coefficient (*r* = 0.345, *P* = 0.006) observed for Cluster 2 than Cluster 1 (*r* = 0.243, *P* = 0.001). Spatial localization of genetic clusters of *Lantana* perhaps also evoked a similar notion of locally adapted individuals across certain geographic regions. Diverse environmental effects have been previously depicted in gene movement in California valley Oak (Sork *et al.* 2010), genetic diversity of Californian Oak (Ortego *et al.* 2012), divergence of a grasshopper (Knowles *et al.* 2007), genetic structure of a Mediterranean plant (Temunovic *et al.* 2012) and genome-wide association to a fine-scale level of eco-heterogeneity in *Biscutella laevigata* (Parisod and Christin 2008).

The presence of isolation by ED pattern, the patchy distribution of genetic clusters and the non-random gene flow evoke the notion of local adaptation. However, our inference is constrained by low resolvability of a limited set of markers and a small number of samples at certain locations. In addition, niche separation of the genetic clusters that may represent divergent ecotypes is not well defined. However, our preliminary conclusions open up opportunities for future works that may consist of characterization of additional individuals with genome-wide polymorphism analysis, preferably using genome-scan or next-generation sequencing. In addition, this may also include phenotypic characterization of individuals, and a comparison between *F*_ST_ and *Q*_ST_ to obtain a better understanding of selective effects.

## Conclusions

Evolutionary trajectories of organisms are complex outcomes of several confounding processes. In order to decipher the dynamics, one needs to uncover the probable interplay between them, i.e. the relative contributions of selection, drift and gene flow. The invasion of *L. camara* into India offers such a unique opportunity to unravel possible scenarios. Using molecular markers along with niche modelling, we elucidated the effect of drift, diverse niche requirements and evidence of isolation by ED or local adaptation. Niche models have predicted the potential distribution almost throughout India. The niches of the divergent genetic clusters are not very different but their geographic localization coupled with unequal gene flow may indicate the emergence of divergent ecotypes. This initial evidence is, however, preliminary, thus may be further investigated by characterizing individuals with genome-wide markers to obtain novel insights into dynamics of adaptive evolution and incipient speciation during invasion.

## Sources of Funding

The National Center for Biological Sciences provided facilities and financial support for this study. A.R. is supported by the Postdoctoral fellowship from the Department of Biotechnology, Government of India.

## Contributions by the Authors

A.R. and R.R. jointly designed and conducted the experiments, analysed the data and wrote the manuscript.

## Conflicts of Interest Statement

None declared.

## Supporting Information

The following Supporting Information is available in the online version of this article–

**Table S1.** *Lantana camara* populations sampled and analysed in this study.

**Table S2.** Descriptive diversity statistics (*A*_R_ is allelic richness, *H*_E_ is heterozygosity, *A*_P_ are private alleles) of three populations and combined populations.

**Table S3.** Pairwise *F*_ST_ values (chloroplast loci and nuclear microsatellites) among populations.

**Table S4.** Data sources of variables used for niche modelling study.

**Table S5.** Correlation table of 19 bioclimatic variables (based on 1200 random points all over India).

**Fig. S1.** AUC values of the models for (a) the whole dataset, (b) Cluster 1 and (c) Cluster 2.

**Fig. S2.** Fine-scale spatial genetic structure in *Lantana*. Autocorrelograms showing the spatial autocorrelation coefficient ‘*r*’ as a function of environmental distance class for (a) Cluster 1 and (b) Cluster 2. 95 % CI about the null hypothesis of a random distribution and 95 % confidence error bars about ‘*r*’ as determined by bootstrapping. Upper (U) and lower (L) confidence limits of the 95 % CI about the null hypothesis of No spatial structure.

Additional Information

## References

[PLU052C2] Beerli P, Bertorelle G, Bruford MW, Hauffe HC, Rizzoli A, Vernesi C (2009). How to use migrate or why are Markov chain Monte Carlo programs difficult to use?. Population genetics for animal conservation, volume 17 of conservation biology.

[PLU052C3] Beerli P, Palczewski M (2010). Unified framework to evaluate panmixia and migration direction among multiple sampling locations. Genetics.

[PLU052C4] Bhagwat SA, Breman E, Thekaekara T, Thornton TF, Willis KJ (2012). A Battle lost? Report on two centuries of invasion and management of *Lantana camara* L. in Australia, India and South Africa. PLoS ONE.

[PLU052C5] Broughton S (2000). Review and evaluation of *Lantana* biocontrol programs. Biological Control.

[PLU052C6] Day MD, Wiley CJ, Playford J, Zalucki MP (2003). Lantana: current management status and future prospects.

[PLU052C7] Duchesne P, Turgeon J (2009). FLOCK: a method for quick mapping of admixture without source samples. Molecular Ecology Resources.

[PLU052C8] Duggin JA, Gentle CB (1998). Experimental evidence on the importance of disturbance intensity for invasion of *Lantana camara* L. in dry rainforest-open forest ecotones in northeastern NSW, Australia. Forest Ecology and Management.

[PLU052C9] Elith J, Phillips SJ, Hastie T, DudIk M, Chee YE, Yates CJ (2011). A statistical explanation of MaxEnt for ecologists. Diversity and Distributions.

[PLU052C11] Endler JA (1986). *Natural selection in the wild.*.

[PLU052C12] Funk WC, Cannatella DC, Ryan MJ (2009). Genetic divergence is more tightly related to call variation than landscape features in the Amazonian frogs *Physalaemus petersi* and *P. freibergi*. Journal of Evolutionary Biology.

[PLU052C13] Garcia-Ramos G, Kirkpatrick M (1997). Genetic models of adaptation and gene flow in peripheral populations. Evolution.

[PLU052C14] Garrant D, Kruuk LEB, Wilkin TA, McCleery RH, Sheldon BC (2005). Evolution driven by differential dispersal within a wild bird population. Nature.

[PLU052C15] Gonzales E, Hamrick JL, Smouse PE, Trapnell DW, Peakall R (2010). The impact of landscape disturbance on spatial genetic structure in the guanacaste tree, *Enterolobium cyclocarpum* (Fabaceae). Journal of Heredity.

[PLU052C16] Groot MD, Rebeušek F, Grobelnik V, Govedi M., Šalamun A, Verovnik R (2009). Distribution modelling as an approach to the conservation of a threatened alpine endemic butterfly (Lepidoptera: Satyridae). European Journal of Entomology.

[PLU052C17] Hendry AP, Day T, Taylor EB (2001). Population mixing and the adaptive divergence of quantitative traits in discrete populations: a theoretical framework for empirical tests. Evolution.

[PLU052C18] Heywood JS (1991). Spatial analysis of genetic variation in plant populations. Annual Review of Ecology and Systematics.

[PLU052C19] Hijmans RJ, Cameron SE, Parra JI, Jones PG, Jarvis A (2005). Very high resolution interpolated climate surfaces for global land areas. International Journal of Climatology.

[PLU052C20] Holderegger R, Buehler D, Gugerli F, Manel S (2010). Landscape genetics of plants. Trends in Plant Science.

[PLU052C21] Jensen JL, Bohonak AJ, Kelley ST (2005). Isolation by distance, web service. BMC Genetics.

[PLU052C22] Johnson S (2007). http://www.dpi.nsw.gov.au/_data/assests/pdf_file/0011/216848/Review-of-the-declaration-of-Lantana-species-in-NSW.pdf.

[PLU052C23] Jones FA, Chen J, Weng GJ, Hubbell SP (2005). A genetic evaluation of seed dispersal in the neotropical tree *Jacaranda copaia* (Bignoniaceae). American Naturalist.

[PLU052C24] Kannan R, Shackleton CM, Uma Shaanker R (2012). Reconstructing the history of introduction and spread of the invasive species, *Lantana*, at three spatial scales in India. Biological Invasions.

[PLU052C25] Kawecki TJ, Ebert D (2004). Conceptual issues in local adaptation. Ecology Letters.

[PLU052C26] Knowles LL, Carstens BC, Keat ML (2007). Coupling genetic and ecological-niche models to examine how past population distributions contribute to divergence. Current Biology.

[PLU052C27] Kohli RK, Batish DR, Singh HP, Dogra KS (2006). Status, invasiveness and environmental threats of three tropical American invasive weeds (*Parthenium hysterophorus* L., *Ageratum conyzoides* L., *Lantana camara* L.) in India. Biological Invasions.

[PLU052C28] Koskinen TM, Haugen TO, Primmer CR (2002). Contemporary fisherian life-history evolution in small salmonid populations. Nature.

[PLU052C29] Lee C-R, Mitchell-Olds T (2011). Quantifying effects of environmental and geographical factors on patterns of genetic differentiation. Molecular Ecology.

[PLU052C30] Legendre P, Fortin M-J (2010). Comparison of the Mantel test and alternative approaches for detecting complex multivariate relationships in the spatial analysis of genetic data. Molecular Ecology Resources.

[PLU052C31] Love A, Babu S, Babu CR (2009). Management of *Lantana*, an invasive alien weed, in forest ecosystems of India. Current Science.

[PLU052C32] Lüi X (2011). Quantitative risk analysis and prediction of potential distribution areas of common *Lantana* (*Lantana camara*) in China. Computational Ecology and Software.

[PLU052C33] Manel S, Schwartz MK, Luikart G, Taberlet P (2003). Landscape genetics: combining landscape ecology and population genetics. Trends in Ecology and Evolution.

[PLU052C34] Manel S, Poncet BN, Legendre P, Gugerli F, Holderegger R (2010). Common factors drive adaptive genetic variation at different spatial scales in *Arabis alpina*. Molecular Ecology.

[PLU052C35] Mateo RG, de la Estrella M, Felicísimo AM, Muñoz J, Guisan A (2013). A new spin on a compositionalist predictive modelling framework for conservation planning: a tropical case study in Ecuador. Biological Conservation.

[PLU052C36] Novak SJ (2007). The role of evolution in the invasion process. Proceedings of the National Academy of Sciences of the USA.

[PLU052C37] Ortego J, Riordan EC, Gugger PF, Sork VL (2012). Influence of environmental heterogeneity on genetic diversity and structure in an endemic southern Californian oak. Molecular Ecology.

[PLU052C38] Parisod C, Christin P-A (2008). Genome-wide association to fine-scale ecological heterogeneity within a continuous population of *Biscutella laevigata* (Brassicaceae). New Phytologist.

[PLU052C39] Peakall R, Smouse PE (2006). GenAlEx 6: genetic analysis in Excel. Population genetic software for teaching and research. Molecular Ecology Notes.

[PLU052C40] Peakall R, Ruibal M, Lindenmayer DB (2003). Spatial autocorrelation analysis offers new insights into gene flow in the Australian bush rat, *Rattus Fuscipes*. . Evolution.

[PLU052C41] Pearson RG (2010). Species' distribution modeling for conservation educators and practitioners. Lessons in Conservation.

[PLU052C42] Pease KM, Freedman AH, Pollinger JP, Mccormack JE, Buermann W, Rodzen J, Banks J, Meredith E, Bleich VC, Schaefer RJ, Jones K, Wayne RK (2009). Landscape genetics of California mule deer (*Odocoileus hemionus*): the roles of ecological and historical factors in generating differentiation. Molecular Ecology.

[PLU052C43] Peterson AT (2003). Predicting the geography of species' invasions via ecological niche modeling. The Quarterly Review of Biology.

[PLU052C44] Petitpierre B, Kueffer C, Broennimann O, Randin C, Daehler C, Guisan A (2012). Climatic niche shifts are rare among terrestrial plant invaders. Science.

[PLU052C45] Phillips SJ, DudIk M (2008). Modeling of species distributions with Maxent: new extensions and a comprehensive evaluation. Ecography.

[PLU052C46] Postma E, Van Noordwijk AJ (2005). Gene flow maintains a large genetic difference in clutch size at a small spatial scale. Nature.

[PLU052C47] Prentis PJ, Wilson JRU, Dormontt EE, Richardson DM, Lowe AJ (2008). Adaptive evolution in invasive species. Trends in Plant Science.

[PLU052C49] Ramírez-Valiente JA, Lorenzo Z, Soto A, Valladares F, Gil F, Aranda I (2010). Natural selection on cork oak: allele frequency reveals divergent selection in cork oak populations along a temperature cline. Evolutionary Ecology.

[PLU052C50] Ray A, Quader S (2013). Genetic diversity and population structure of *Lantana camara* in India indicates multiple introductions and gene flow. Plant Biology.

[PLU052C51] Ray A, Sumangala RC, Ravikanth G, Uma Shaanker R, Quader S (2012). Isolation and characterization of polymorphic microsatellite loci from the invasive plant *Lantana camara* L. Conservation Genetics Resources.

[PLU052C52] Rodriguez JP, Brotons L, Bustamante J, Seoane J (2007). The application of predictive modelling of species distribution to biodiversity conservation. Diversity and Distributions.

[PLU052C53] Slatkin M (1985). Gene flow in natural populations. Annual Review of Ecology, Evolution and Systematics.

[PLU052C54] Smouse PE, Peakall R (1999). Spatial autocorrelation analysis of individual multiallele and multilocus genetic structure. Heredity.

[PLU052C55] Sork VL, Davis FW, Westfall R, Flint A, Ikegami M, Wang H, Grivet D (2010). Gene movement and genetic association with regional climate gradients in California valley oak (*Quercus lobata* Née) in the face of climate change. Molecular Ecology.

[PLU052C56] Stirton CH, Annecke DP (1978). Some thoughts on the polyploid complex *Lantana camara* L. (Verbenaceae). Proceedings of the second national weeds conference.

[PLU052C57] Swarbrick JT, Wilson BB, Hannan-Jones MA, Panetta FD, Groves RH, Shephard RCH (1998). *Lantana camara* L. In.

[PLU052C58] Taylor S, Kumar L (2012). Sensitivity analysis of CLIMEX parameters in modelling potential distribution of *Lantana camara* L. PLoS ONE.

[PLU052C59] Taylor S, Kumar L (2013). Potential distribution of an invasive species under climate change scenarios using CLIMEX and soil drainage: a case study of *Lantana camara* L. in Queensland, Australia. Journal of Environmental Management.

[PLU052C60] Temunovic M, Franjic J, Satovic Z, Grgurev M, Frascaria-Lacoste N, Fernandez-Manjarres JF (2012). Environmental heterogeneity explains the genetic structure of continental and mediterranean populations of *Fraxinus angustifolia* Vahl. PLoS ONE.

[PLU052C61] Thakur ML, Ahmad M, Thakur RK (1992). *Lantana* weed (*Lantana camara* var. *aculeata* Linn.) and its possible management through natural insect pests in India. Indian Forester.

[PLU052C70] Tiefelsdorf M (2000). Modeling Spatial Processes: The Identification and Analysis of Spatial Relationships in Regression residuals by Means of Moran's I.

[PLU052C62] Torres E, Iriondo JM, Escudero A, Perez C (2003). Analysis of within-population spatial genetic structure in *Antirrhinum microphyllum* (Scrophulariaceae). American Journal of Botany.

[PLU052C63] Urbina-Cardona JN, Loyola RD (2008). Applying niche-based models to predict endangered-hylid potential distributions: are neotropical protected areas effective enough?. Tropical Conservation Science.

[PLU052C64] Vaclavik T, Meentemeyer RK (2012). Equilibrium or not? Modelling potential distribution of invasive species in different stages of invasion. Diversity and Distributions.

[PLU052C65] Wang IJ, Summers K (2010). Genetic structure is correlated with phenotypic divergence rather than geographic isolation in the highly polymorphic strawberry poison-dart frog. Molecular Ecology.

[PLU052C66] Warren DL, Glor RE, Turelli M (2010). ENMTools: a toolbox for comparative studies of environmental niche models. Ecography.

[PLU052C67] Welk E, Schubert K, Hoffmann MH (2002). Present and potential distribution of invasive garlic mustard (*Alliaria petiolata*) in North America. Diversity and Distributions.

[PLU052C68] Zalba SM, Sonaglioni MI, Compagnoni CA, Belenguer CJ (2000). Using a habitat model to assess the risk of invasion by an exotic plant. Biological Conservation.

[PLU052C69] Zhu L, Sun OJ, Sang W, Li Z, Ma K (2007). Predicting the spatial distribution of an invasive plant species (*Eupatorium adenophorum*) in China. Landscape Ecology.

