# Supplementary material for: Rapid divergence of ecotypes of an invasive plant
Source: AoB Plants. 2014 Sep 1;6:plu052. doi: 10.1093/aobpla/plu052 (PMC4215188; doi:10.1093/aobpla/plu052)
Supplement: Additional Information [file supp_6_plu052_index.html]

Rapid divergence of ecotypes of an invasive plant — Additional Information 

# Rapid divergence of ecotypes of an invasive plant

## Additional Information

Additional Information

**Files in this Data Supplement:**

- Supplementary Figures - docx file
- Supplementary Table 1 - docx file
- Supplementary Table 2 - doc file
- Supplementary Table 3 - docx file
- Supplementary Table 4 - doc file
- Supplementary Table 5 - doc file
