# Supplementary material for: Rapid divergence of ecotypes of an invasive plant
Source: AoB Plants. 2014 Sep 1;6:plu052. doi: 10.1093/aobpla/plu052 (PMC4215188; doi:10.1093/aobpla/plu052)

Figure S1a-c: AUC values of the models for a) the whole dataset, b) cluster 1, and c) cluster 2

Figure S2: Fine-scale spatial genetic structure in *Lantana*. Autocorrelograms showing the spatial autocorrelation coefficient ‘r ‘as a function of environmental distance class for a) cluster 1 and b) cluster 2. 95% CI about the null hypothesis of a random distribution and 95% confidence error bars about ‘r’ as determined by bootstrapping


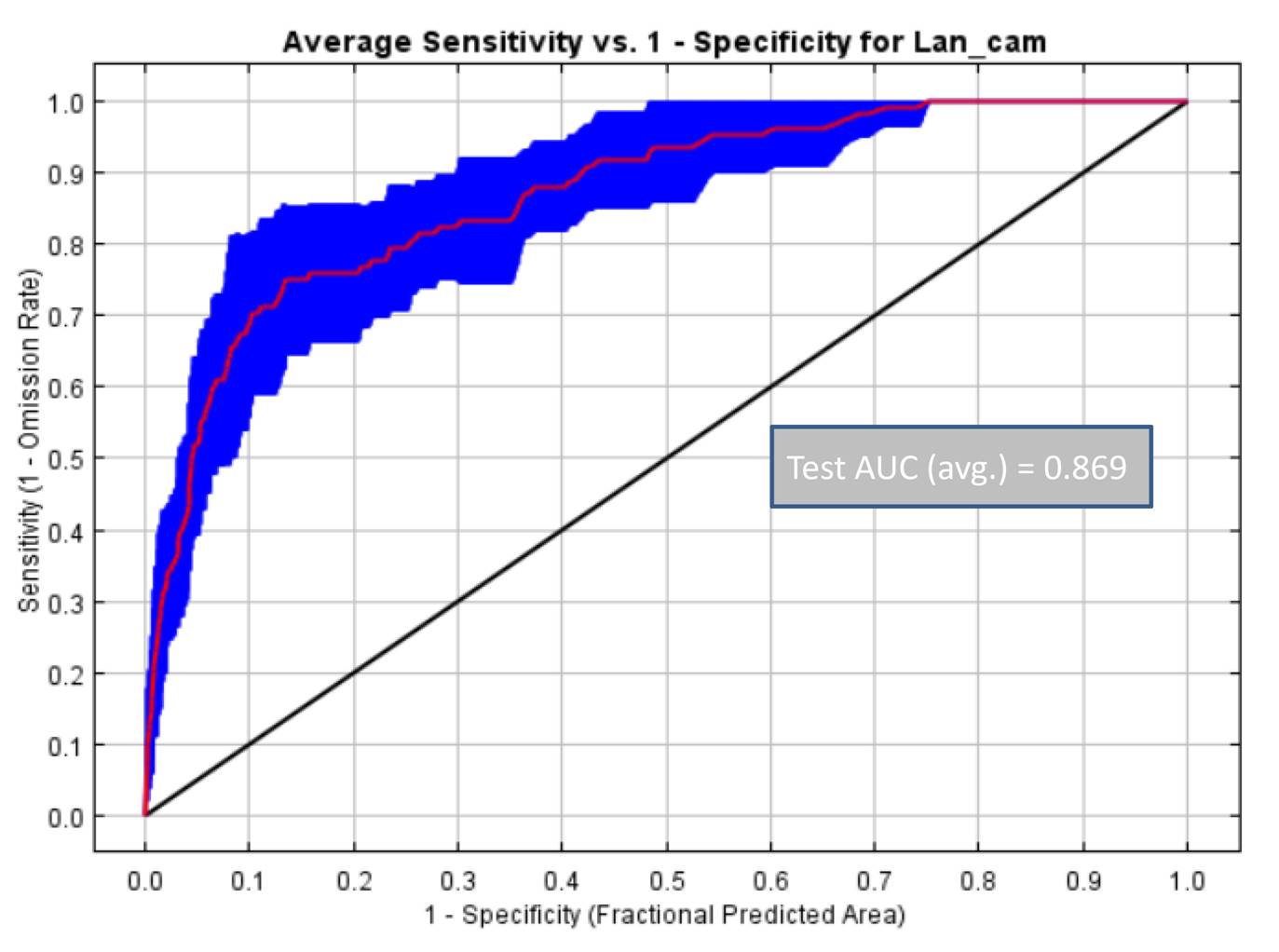


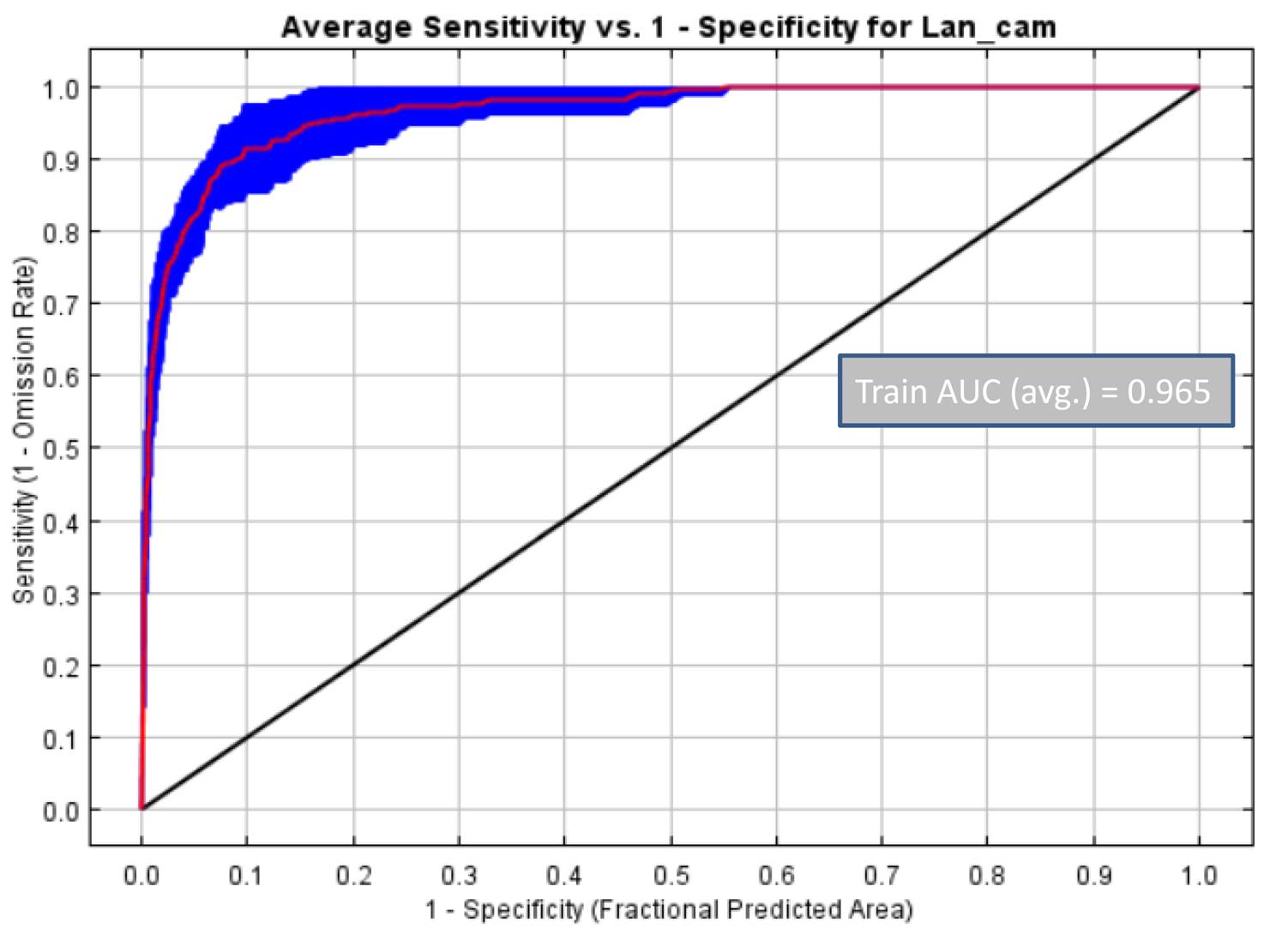


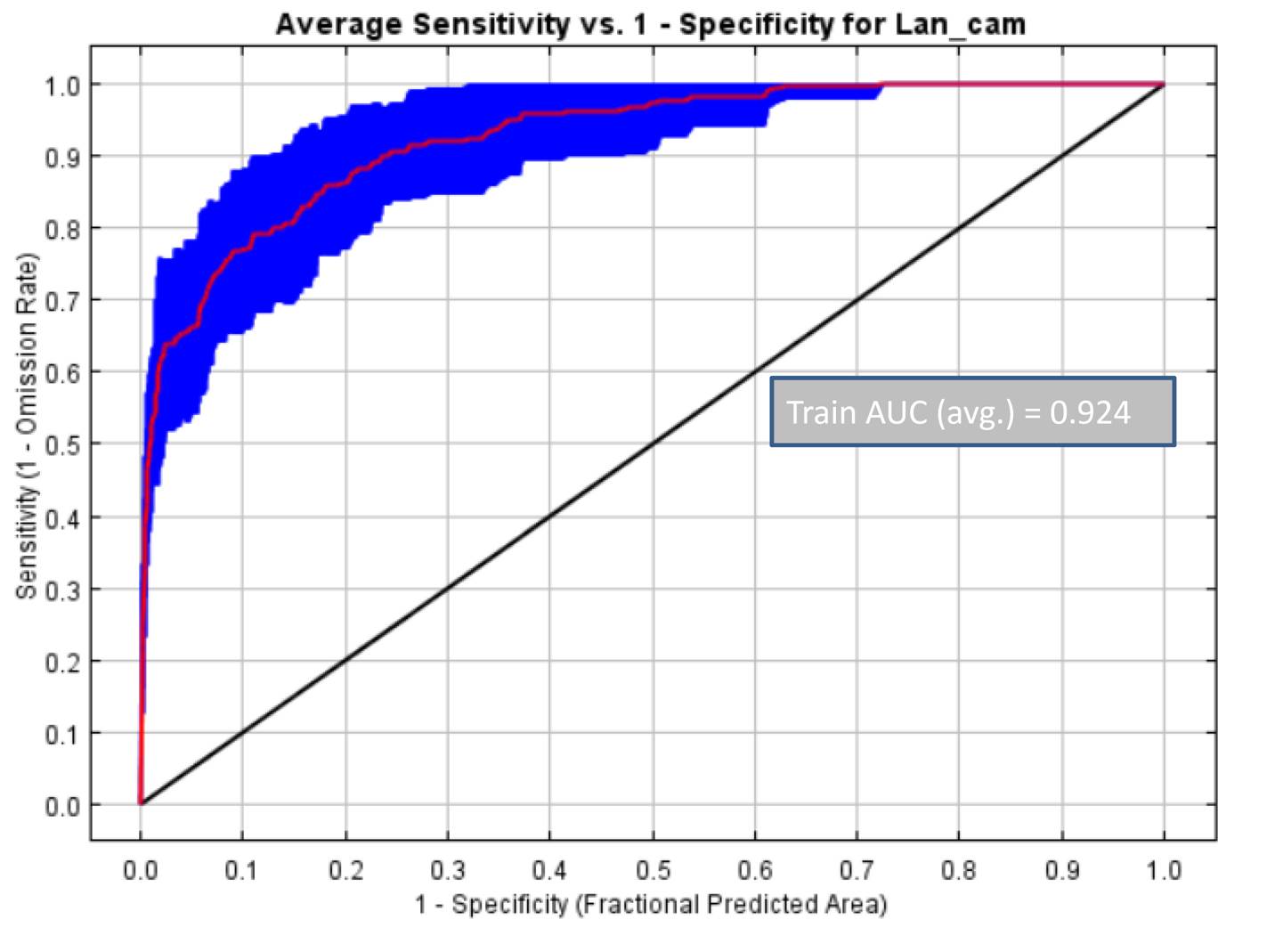


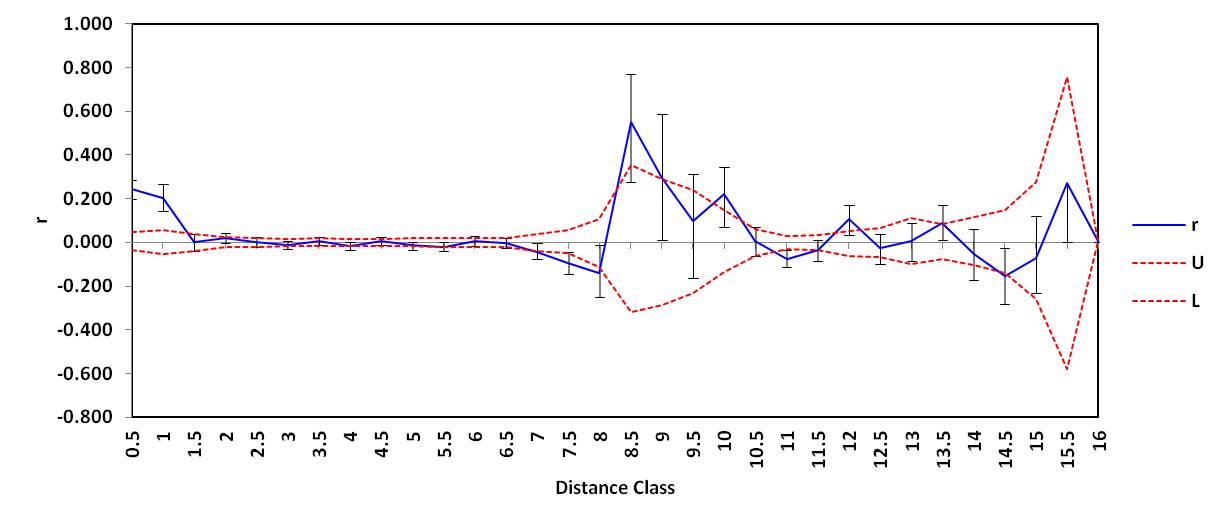


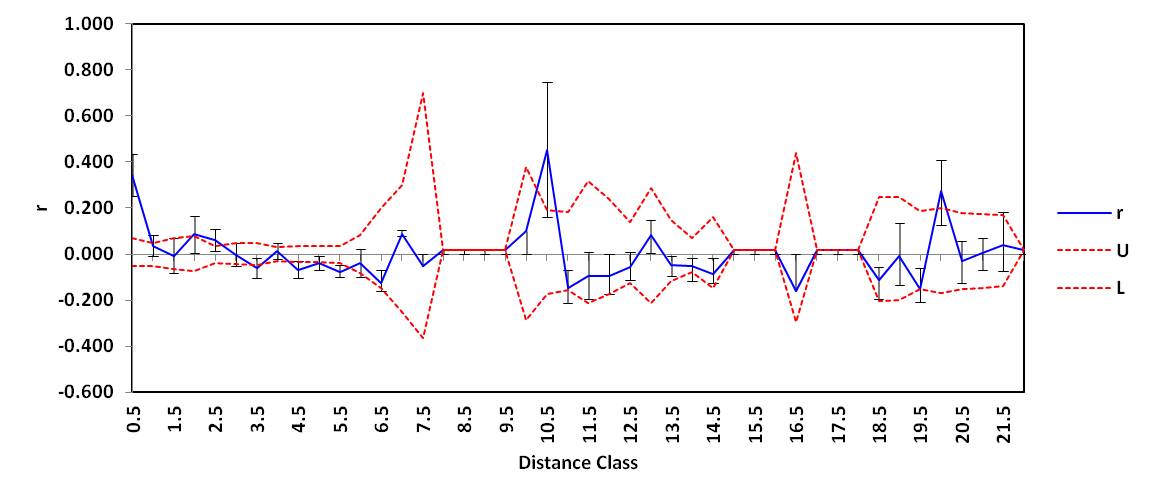

Supplement: Additional Information [file supp_plu052_plu052supp_figs.docx]
