# Supplementary material for: Rapid divergence of ecotypes of an invasive plant
Source: AoB Plants. 2014 Sep 1;6:plu052. doi: 10.1093/aobpla/plu052 (PMC4215188; doi:10.1093/aobpla/plu052)
Supplement: Additional Information [file supp_plu052_plu052supp_table1.docx]

**Table S1:** *Lantana camara* populations sampled and analyzed in this study

| Populations | State/ Province | Locality | longitude | Latitude | Number of individuals analyzed | |
| --- | --- | --- | --- | --- | --- | --- |
|  |  |  |  |  | Chloroplast loci (n) | Nuclear Microsatellite loci (n) |
| Eastern | West Bengal | Kolkata | 88°24’06.71” E | 22°30’39.46”N | 26 | 29 |
|  |  | Nadia | 88°29'43.51"E | 23°22'38.00"N | 3 | 3 |
|  |  | Purulia | 85°58'20.66"E | 23°17'10.43"N | 3 | 3 |
|  |  | Bankura | 86°57'53.64"E | 23°23'29.61"N | 4 | 5 |
|  |  | Shiliguri | 88°28' 6.06" E | 26° 51' 58.36"N | 3 | 3 |
|  | Orissa | Bhubaneswar | 85°50'24.00"E | 20°16'12.00"N | 4 | 4 |
|  |  | Mayurbhanj | 86°24'57.32"E | 21°53'44.30"N | - | 1 |
|  |  | Raygada | 83°24'53.99"E | 19°10'0.67"N | - | 1 |
|  | Assam | Jorhat | 94°12'26.20"E | 26°45'13.28"N | 3 | 3 |
|  | Tripura | Agartola | 91°16'59.54"E | 23°50'9.35"N | 5 | 6 |
|  | Meghalaya | Nongpoh | 91°53'3.58"E | 25°54'0.18"N | 1 | 1 |
|  | Arunachal | Itanagar | 93°41'17.67"E | 27° 6'8.97"N | 1 | 1 |
| Northern | Delhi | Delhi | 77°19'11.99"E | 28°29'23.99"N | 3 | 3 |
|  | HP | Mandala | 76° 52' 46.13" | 30° 53' 59.53" | 2 | 11 |
|  | UP | Varanasi | 82°59'31.49"E | 25°17'1.69"N |  | 3 |
|  |  | Jhansi | 78°32.574E | 25°30.217'N | 3 | 2 |
|  | UK | Ramnagar | 78°51'47.00"E | 29°35'31.09"N | 3 | 7 |
|  |  | Haridwar | 78° 3'8.89"E | 30° 7'47.14"N | 1 | 2 |
|  |  | Dehradun | 77°58'30.56"E | 30°17'0.38"N | 14 | 15 |
|  |  | Okhimath | 79° 4'42.69"E | 30°31'27.85"N | 3 | 3 |
|  | MP | Bhopal | 77°24'28.06"E | 23°16'8.42"N | 11 | 8 |
|  |  | Ujjain | 75°51'2.15"E | 22°37'24.13"N | 3 | 7 |
|  | Rajasthan | Udaypur | 73°48.533E | 24°32.084N | 15 | 15 |
|  |  | Kota | 75°49'48.00"E | 25°10'48.00"N | 6 | 6 |
|  | Gujarat | Junagrah | 70°45'26.13"E | 21° 6'23.95"N | 1 | 5 |
| Southern | Karnataka | Bangalore | 77°34’29.69”N | 12°56’31.74”N | 6 | 7 |
|  |  | Kodagu | 76°28’52.54”N | 12°51’18.45”N | 9 | 10 |
|  |  | Shimoga | 75°20'52.66"E | 14°9'49.25" | 3 | 3 |
|  |  | Uttar Kannada | 74°23’37.34”N | 14°25’18.26”N | 7 | 10 |
|  | Andhra Pradesh | Vishakhapatnam | 83°14'57.08"E | 17°44'13.00"N | 6 | 6 |
|  |  | Rishi Valley | 76°03'36.51"E | 14°27'51.05"N | 3 | 3 |
|  |  | Hyderabad | 78°32'26.12"E | 17°25'17.37"N | 2 | 3 |
|  | Tamilnadu | Valparai | 77°49’E | 10°26`N | 10 | 11 |
|  | Kerala | Kollam | 76°38'15.47"E | 8°52'54.92"N | 4 | 4 |
|  |  | Munnar | 77°14'41.28"E | 10°7'19.52"N | 2 | 2 |
|  | Maharshtra | Nagpur | 79°13'38"E | 20°55'9.22" N | 6 | 7 |
|  |  | Pune | 73°48'14.23"E | 18°27'25.85"N | 3 | 5 |
| Total | | | | | 179 | 218 |
