# Supplementary material for: Rapid divergence of ecotypes of an invasive plant
Source: AoB Plants. 2014 Sep 1;6:plu052. doi: 10.1093/aobpla/plu052 (PMC4215188; doi:10.1093/aobpla/plu052)
Supplement: Additional Information [file supp_plu052_plu052supp_table2.doc]

|  |  |  |  |  |  |  |  |  |  |  |  |  |  |  |  |  |  |  |  |
| --- | --- | --- | --- | --- | --- | --- | --- | --- | --- | --- | --- | --- | --- | --- | --- | --- | --- | --- | --- |
|  |  |  |  |  |  |  |  |  |  |  |  |  |  |  |  |  |  |  |  |
|  |  |  |  |  |  |  |  |  |  |  |  |  |  |  |  |  |  |  |  |
|  |  |  |  |  |  |  |  |  |  |  |  |  |  |  |  |  |  |  |  |
|  |  |  |  |  |  |  |  |  |  |  |  |  |  |  |  |  |  |  |  |
|  |  |  |  |  |  |  |  |  |  |  |  |  |  |  |  |  |  |  |  |
|  |  |  |  |  |  |  |  |  |  |  |  |  |  |  |  |  |  |  |  |
|  |  |  |  |  |  |  |  |  |  |  |  |  |  |  |  |  |  |  |  |
|  |  |  |  |  |  |  |  |  |  |  |  |  |  |  |  |  |  |  |  |

| Populations | Sample size (n) | Allelic richness (A_R_) | | | | | | Heterozygosity (He) | | | | | | Private alleles (A_p_) | | | | | |
| --- | --- | --- | --- | --- | --- | --- | --- | --- | --- | --- | --- | --- | --- | --- | --- | --- | --- | --- | --- |
|  |  | Lac6 | Lac11 | Lac13 | Lac14 | Phc17 | Phc18 | Lac6 | Lac11 | Lac13 | Lac14 | Phc17 | Phc18 | Lac6 | Lac11 | Lac13 | Lac14 | Phc17 | Phc18 |
| Total | 218 | 11 | 13 | 19 | 19 | 12 | 9 | 0.5685 | 0.7119 | 0.8167 | 0.8444 | 0.4045 | 0.699 | 4 | 9 | 6 | 3 | 2 | 3 |
| Eastern | 60 | 9 | 9 | 15 | 18 | 11 | 5 | 0.5403 | 0.7502 | 0.829 | 0.8472 | 0.4765 | 0.6508 | 2 | 5 | 2 | 3 | 1 | 1 |
| Northern | 87 | 8 | 8 | 17 | 16 | 7 | 7 | 0.5393 | 0.719 | 0.7994 | 0.8615 | 0.2868 | 0.7561 | 2 | 4 | 4 | 0 | 0 | 1 |
| Southern | 71 | 4 | 4 | 10 | 14 | 11 | 6 | 0.6140 | 0.6511 | 0.7946 | 0.8001 | 0.4670 | 0.5883 | 0 | 0 | 0 | 0 | 1 | 1 |

**Table S2:** Descriptive diversity statistics (A_R_= Allelic richness, H_E_= Heterozygosity, A_P_=Private Alleles) of three populations and combined populations
