# Supplementary material for: Rapid divergence of ecotypes of an invasive plant
Source: AoB Plants. 2014 Sep 1;6:plu052. doi: 10.1093/aobpla/plu052 (PMC4215188; doi:10.1093/aobpla/plu052)
Supplement: Additional Information [file supp_plu052_plu052supp_table3.docx]

**Table-S 3:** Pairwise F_ST_ values (chloroplast loci and nuclear microsatellites) among populations

|  | Chloroplast loci | | | Nuclear Microsatellites | | |
| --- | --- | --- | --- | --- | --- | --- |
|  | Eastern | Northern | Southern | Eastern | Northern | Southern |
| Eastern | - | - | - | - | - | - |
| Northern | -0.009 | - | - | 0.00218 | - | - |
| Southern | 0.056* | 0.052* | - | 0.0357* | 0.0383* | - |

*Significant, P<0.05
