# Supplementary material for: Rapid divergence of ecotypes of an invasive plant
Source: AoB Plants. 2014 Sep 1;6:plu052. doi: 10.1093/aobpla/plu052 (PMC4215188; doi:10.1093/aobpla/plu052)
Supplement: Additional Information [file supp_plu052_plu052supp_table4.doc]

Table S4.

| Variable type | Resolution | Source |
| --- | --- | --- |
| Climate  Altitude  Landcover  Soil Type  Soil moisture  Solar radiation | 1km^2^  90 m^2^  1km^2^  ~55km^2^  10 km^2^ | [http://www.worldclim.org](http://www.worldclim.org/)  [http://srtm.csi.cgiar.org](http://www.cgiar-csi.org/2010/03/108/uot;http:/srtm.csi.cgiar.org)  <http://www.diva-gis.org/gdata>  www.fao.org/geonetwork/srv/en/main.home  <http://climate.geog.udel.edu/~climate/html_pages/download_whc150_ts2.html>  <http://mnre.gov.in/sec/solar-assmnt.htm> |
