# Supplementary material for: Rapid divergence of ecotypes of an invasive plant
Source: AoB Plants. 2014 Sep 1;6:plu052. doi: 10.1093/aobpla/plu052 (PMC4215188; doi:10.1093/aobpla/plu052)
Supplement: Additional Information [file supp_plu052_plu052supp_table5.doc]

Table S5: Correlation table of 19 bioclimatic variables (based on 1200 random points all over India)
